# The Linac Coherent Light Source

**DOI:** 10.1107/S1600577515005196

**Published:** 2015-04-21

**Authors:** William E. White, Aymeric Robert, Mike Dunne

**Affiliations:** a Linac Coherent Light Source, SLAC National Accelerator Laboratory, 2575 Sand Hill Road, Menlo Park, CA 94025, USA

**Keywords:** FEL, X-ray, Linac Coherent Light Source

## Abstract

The Linac Coherent Light Source (LCLS) at the SLAC National Accelerator Laboratory was the first hard X-ray free-electron laser (FEL) to operate as a user facility. After five years of operation, LCLS is now a mature FEL user facility. Our personal views about opportunities and challenges inherent to these unique light sources are discussed.

## Abstract 2

The present status of the Linac Coherent Light Source as a user facility is presented. Opportunities and challenges as well as the scientific impact of X-ray free-electron lasers are discussed.

## 1. Introduction   

In 1962, a team of physicists from Stanford began work on project ‘Monster’, an ambitious plan to construct the largest linear accelerator (linac) ever built. While it is not clear how long they thought the completed project would remain relevant, it is almost certain that this team of physicists did not visualize the amazing transformation this machine would undergo after five decades of service to the field of high-energy physics. The use of the linac to drive a revolutionary light source would surely have been hard to predict at a time ten years before the concept of using an accelerator as an X-ray light source was realized at the SPEAR synchrotron at the Stanford Linear Accelerator Center, SLAC.

This resurrection began in spirit at the Workshop on Fourth Generation Light Sources in February 1992, where Claudio Pellegrini and others presented calculations showing that the SLAC linac could be used as the engine to drive an X-ray free-electron laser (FEL) (Pellegrini, 1992 ▶). Later in October that year, William Spicer, Hermann Winick and John Arthur held a Workshop on Scientific Applications of Short Wavelength Coherent Light Sources to discuss the science that could be realized with such a machine. Throughout that decade, multiple workshops led to increased momentum and, in 1997, the US Department of Energy (DOE) formed a committee to investigate future light sources. By the turn of the century, the possibility of a user facility was regarded as credible, and in 2001 John Galayda joined SLAC to direct the Linac Coherent Light Source (LCLS) construction project. Eight years later, in April 2009, a group of scientists led by Paul Emma demonstrated the first lasing of a hard X-ray FEL (Emma *et al.*, 2010 ▶). A review of the history of X-ray free-electron lasers which led to the construction of LCLS is given by Pellegrini (2012) ▶.

The excitement leading up to first light, combined with how well the machine worked from the very beginning, fueled efforts to generate the first science output as rapidly as possible. The LCLS project included the construction of one scientific instrument, the Atomic, Molecular and Optical (AMO) instrument in the first hutch of the newly constructed Near Experimental Hall, to support this goal. It led to the first user experiments being performed in October 2009.

## 2. Facility description   

**Figure 1:**
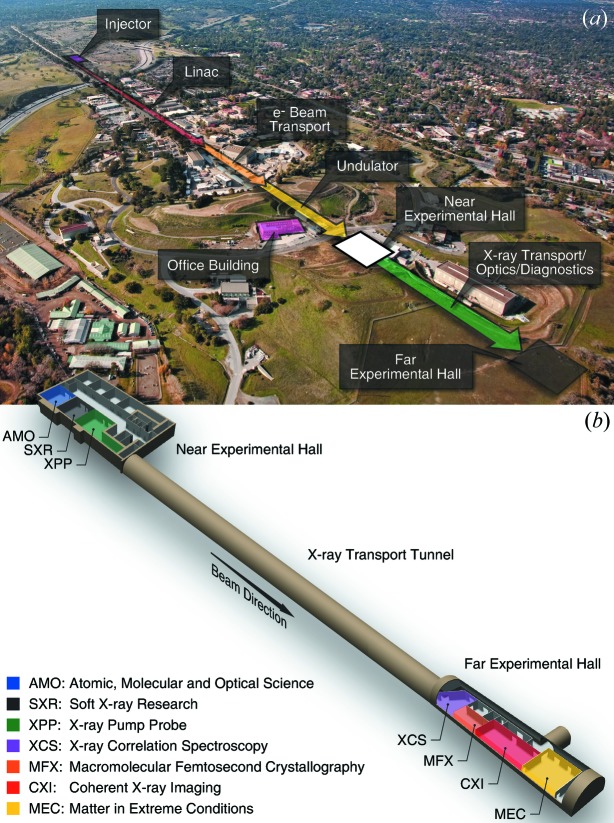

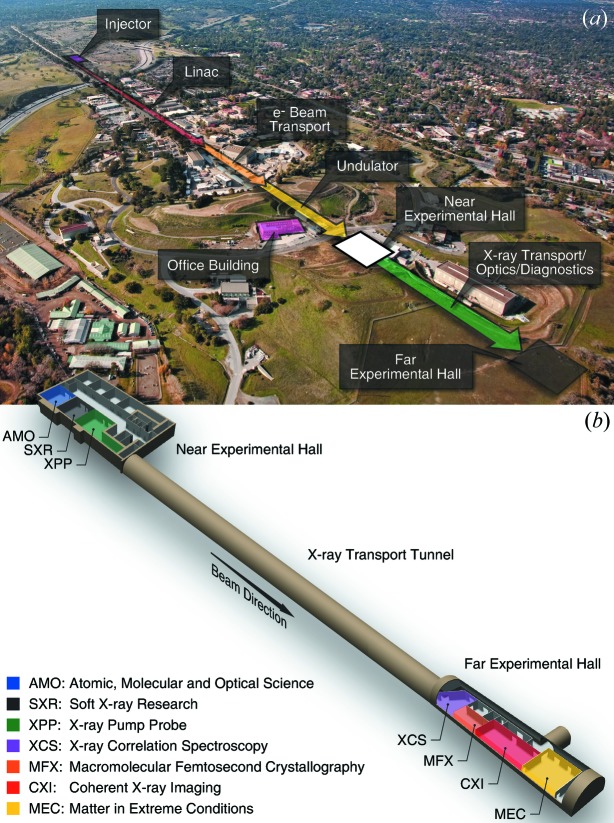
Description of the LCLS facility on the SLAC National Accelerator Laboratory site. (*a*) Annotated aerial view highlighting the various areas of LCLS, including the injector, linac, undulator and experimental halls. (*b*) Details of the location of the LCLS experimental hutches in the experimental halls.

The LCLS construction project delivered an experimental facility that consists of three experimental hutches in the Near Experimental Hall and three more experimental hutches in the Far Experimental Hall, connected by a 200 meter-long transport tunnel, as described in Fig. 1 ▶(*a*). The instruments in the remaining five hutches were completed over a period of 40 months. This was supported by a dedicated construction project funded by DOE (known as LUSI–LCLS Ultrafast Science Instruments) that funded the construction of three instruments (XPP, XCS, CXI), coupled to a separate consortium funding the SXR instrument. More recently, the MEC instrument was jointly funded by the Fusion Energy Sciences and Basic Energy Sciences programs in DOE, leading to completion of the current suite of LCLS instruments in January 2013. The location of all LCLS instruments is shown in Fig. 1 ▶(*b*).

Each of these instruments was built in response to the report ‘The First Experiments’ (Shenoy & Stohr, 2003 ▶), detailing the conclusions from a group of scientists led by Gopal Shenoy and Joachim Stohr, which highlights the initial breadth of scientific interest and excitement in the unique capabilities of LCLS.

The LCLS instruments are optimized for the soft X-ray (250 eV–2 keV) or the hard X-ray (3 keV–11 keV) range that can be provided by the first harmonic of the undulators. Some can also use the third harmonic of the spectrum. Their configuration can be summarized as follows, with each of them offering capabilities to study many areas of science using the unique X-ray FEL beam properties:

AMO – Atomic, Molecular and Optical instrument utilizes a highly focused and intense soft X-ray beam and offers the possibility to perform imaging, time-resolved spectroscopy and high power density studies (Ferguson *et al.*, 2015 ▶).

SXR – Soft X-ray Research instrument utilizes FEL radiation to study spectroscopically various fields of science such as ultrafast chemistry, highly correlated materials, magnetism and surface science (Dakovski *et al.*, 2015 ▶).

XPP – X-ray Pump–Probe instrument uses femtosecond time-resolved hard X-ray diffraction, scattering and spectroscopy techniques to study the photo-induced dynamics of physical, chemical and biological systems at the atomic scale (Chollet *et al.*, 2015 ▶).

XCS – X-ray Correlation Spectroscopy instrument exploits the unique coherent properties of the LCLS hard X-ray beam, by using coherent X-ray scattering techniques. This allows investigating structural and dynamical phenomena in complex and disordered systems down to the atomic scale (Alonso-Mori *et al.*, 2015*a* ▶).

CXI – Coherent X-ray Imaging instrument takes advantage of its intense micrometer and sub-micrometer size focused hard X-ray beams for primarily forward scattering studies of very small weakly scattering objects. CXI is used, for example, to perform protein crystallography studies of small crystals, X-ray/matter interaction studies under intense X-ray illumination and is also the platform to develop molecular imaging by means of coherent diffractive imaging using hard X-rays (Liang *et al.*, 2015 ▶).

MEC – Matter in Extreme Conditions instrument provides the ability to use high power optical laser beams to generate matter at extreme temperatures and pressures, allowing studies of transient phenomena that are highly relevant to fields as diverse as astrophysics, planetary physics, geophysics and plasma physics (Nagler *et al.*, 2015 ▶).

Significant development has followed ever since to expand the capacity of the facility, including techniques for multiplexing instruments (Feng *et al.*, 2015 ▶), fielding serial samples on a single beamline (Boutet *et al.*, 2015 ▶) and the upcoming construction of a seventh instrument for macromolecular femtosecond crystallography (MFX) in the Far Experimental Hall, as indicated in Fig. 1 ▶(*b*).

## 3. Opportunities and challenges   

The characteristics of X-ray FEL pulses present an interesting duality of challenges and opportunities. The peak brightness, short pulse length, full transverse coherence and the broad but stochastically varying spectrum can all be seen as both problematic and enabling. For example, the intensity of the focused X-ray beam can result in irreversible sample damage from a single pulse, rendering further measurements impossible. However, the brightness that is responsible for this damage can be combined with the short pulse duration to provide a measurement approach that allows meaningful signals to be obtained within that single shot yielding substantial information prior to destruction. This opens up the potential to study samples that are precluded from investigation using conventional approaches. The so-called ‘diffract-before-destroy’ technique is a prime example of this (Neutze *et al.*, 2000 ▶).

This, however, is a fundamentally different paradigm to the approaches and techniques adopted at storage ring light sources, and has forced a complete re-evaluation of all aspects of the experiments from the theoretical assessment of what physical properties can be investigated, through preparation and delivery of samples, to development of wholly new measurement and detection techniques, and finally the data extraction and analysis at the back end of the experiment. In some ways, such systems are hybrids of synchrotron measurements with some typical aspects of high-power laser plasma physics experiments. Overall, with hindsight, it is clearly a misnomer to classify an X-ray FEL as a ‘fourth-generation light source’. Quite simply, it is a wholly new class of experimental tool and needs to be considered as such when contemplating science opportunities, experimental configurations, facility operations and data interpretation. By adopting a new mindset, we can realize the unique potential of LCLS and other emerging FEL facilities, and avoid the pitfall of incremental approaches or comparisons with storage ring facilities.

The intrinsic pulsed nature of the FEL sources suits the requirements of a range of broadly enabling experimental techniques such as pump–probe. In these experiments, an excitation (ultrafast optical, THz, high pulsed magnetic field, *etc.*) is triggered with a controlled degree of synchronization with each FEL pulse, allowing studies of both ultrafast phenomena and stroboscopic evolution over extended timescales of systems that require an extremely bright probe source. A further example is where the high peak brightness of LCLS has allowed the high-power laser community to merge with the traditional X-ray world, opening up new opportunities in investigating transient behaviors of ‘matter in extreme conditions’ (Nagler *et al.*, 2015 ▶).

In considering how best to exploit the incredible peak brightness, along with short temporal duration, coherence and potential high degree of control over all these pulse characteristics, we can access entirely new avenues of investigation. This requires experimental solutions that can cope with the fact that every shot is different and there can be substantial jitter in the energy spectrum, as well as temporal and spatial properties of the X-ray beam. This ‘shot-by-shot’ technique is eventually further complicated by the need for continuous refreshing of the sample and the fact that the samples may not be reproducible at the length-scales of interest. The consequences of such challenges include:

(i) A broad suite of information needs to be recorded for every single shot (including machine parameters and multiple diagnostic measurements that are required in order to properly interpret a single-shot event), which on a facility stretching across several kilometers is challenging in terms of data acquisition, synchronization and retrieval.

(ii) The continuous development and integration of new diagnostics to characterize the properties of every single shot (Moeller *et al.*, 2015 ▶), but also the development of more elaborate ways to address more complex forms of jitter, such as the focal properties (Sikorski *et al.*, 2015 ▶) and spectra of the FEL beam (Zhu *et al.*, 2012 ▶).

(iii) The emergence of experiments requiring several concurrent techniques in order to fully understand the process probed by individual shots. This is, in particular, the case with the application and development of spectroscopy techniques (Alonso-Mori *et al.*, 2015*b* ▶) in combination with other diffuse scattering or diffraction techniques.

(iv) A whole suite of laser systems are needed across all LCLS instruments (Minitti *et al.*, 2015 ▶), requiring the delivery of reliably tailored optical pulses in terms of wavelength, power, pulse duration, spatial profile and repetition rate, with precise control of time delay with respect to the LCLS pulses. This includes the development of laser-based secondary sources of excitation such as THz radiation that are requested for specific experiments (Turner *et al.*, 2015 ▶).

(v) New imaging detectors are needed in order to take full advantage of these sources, both for soft-X-ray and hard-X-ray experiments. Recording all scattered photons that emerge within a sub-100 fs pulse is a real challenge. A review of the LCLS detector program is given by Blaj *et al.* (2015 ▶).

(vi) Imaging detectors developed specifically for FEL sources also require very specific treatment in terms of signal correction (cross-talk, nonlinearity, *etc.*) in order to successfully lead to correct data interpretation (van Driel *et al.*, 2015 ▶).

(vii) New sample injection and delivery mechanisms are needed to exploit the full capability of the high repetition rate and brightness, including in-air operation.

(viii) The development of new data recording and data mining techniques are required for the output generated by detectors at rates from 10 to 10^6^ Hz.

In adapting to science at LCLS, some strong and well-established scientific communities, such as the protein crystallography (PX) community, have had to develop wholly new approaches to their field. They had to return to the fundamentals of developing new measurement techniques, having been used to a high degree of automation on third-generation machines. This required a strong R&D phase, where every aspect of the experiment requires significant attention, and from which complementary philosophies of the most productive approach have emerged. For example, there are substantial trade-offs and differences of opinion between the future impact of macromolecular crystallography (derivative of goniometer-based techniques used on synchrotrons) and the emerging field of serial femtosecond crystallography (Schlichting, 2015 ▶), with the ultimate goal of diffraction from single particles entrained in a liquid jet. Such debates are the lifeblood of newly emerging fields, and illustrate the healthy evolution that is driven by a major change in this new type of light sources, such as LCLS.

The baseline operation of LCLS as a self-amplified stimulated emission (SASE) beam originating from a stochastic process means that each pulse can present very different temporal, spectral and spatial properties. As such, a wide range of schemes is being developed to control the FEL pulses. In parallel, many diagnostics are being developed to measure these differences at the single-shot level in order to allow correction and correlation in the data analysis. Examples include:

(i) Self-seeding (and ultimately external seeding) to control the longitudinal coherence length and thus the monochromaticity of the output light (Amann *et al.*, 2012 ▶; Ratner *et al.*, 2015 ▶).

(ii) Control of the bunch charge and envelope to deliver ultra-short pulses, with durations as short as a few femto­seconds.

(iii) Undulators to manipulate the polarization.

(iv) Control of the spatial and timing jitter, along with precision measurements of the pulse envelope using techniques such as X-ray Transverse CAVity (XTCAV) (Ding *et al.*, 2011 ▶).

(v) In-line spectrometers to measure the spectrum of a single shot (Zhu *et al.*, 2012 ▶).

(vi) Generation of multi-bunches, providing color separation at the percent level (and ultimately at the multi-100 eV level to provide platforms for studying multiple elements simultaneously).

## 4. Scientific impact   

The associated collection of papers in this journal focuses on the technical capabilities of the LCLS instruments. However, just five years into the operational phase of this new facility, it is essential to note that their scientific impact has already been profound. Details of this are to be explored in a separate article (Bostedt *et al.*, 2015 ▶), but the breadth of research topics includes:

(i) Life sciences: structure measurements of photosystem I *via* femtosecond nanocrystallography (Chapman *et al.*, 2011 ▶); the first coherent diffraction images of a single virus particle with an X-ray FEL (Seibert *et al.*, 2011 ▶); high-resolution protein structure determination (Boutet *et al.*, 2012 ▶); and the first *de novo* structure determination from an X-ray laser (Barends *et al.*, 2014 ▶).

(ii) Plasma physics: creation and diagnosis of solid density plasmas (Vinko *et al.*, 2012 ▶).

(iii) Magnetism: single-shot imaging of nanoscale ferromagnetic order *via* resonant X-ray holography (Wang *et al.*, 2012 ▶); the first study of the dynamics of laser-driven switching in magnetic materials with chemical selectivity (Graves *et al.*, 2013 ▶); and the demonstration of the possibility of manipulating atomic scale magnetic structure with the electric field of light on a sub-picosecond timescale (Kubacka *et al.*, 2014 ▶).

(iv) Chemistry: investigation of the photo-induced spin crossover transition in metal transition complexes by time-resolved femtosecond X-ray absorption (Lemke *et al.*, 2013 ▶) and fluorescence (Zhang *et al.*, 2014 ▶) spectroscopy.

(v) Materials science: fractal morphology, imaging and mass spectrometry of single aerosol particles in flight (Loh *et al.*, 2012 ▶); the first observation of the three-dimensional structural response of nanocrystal lattice dynamics following laser excitation (Clark *et al.*, 2013 ▶); the measurement of the phonon dispersion of germanium with time-resolved X-ray diffuse scattering (Trigo *et al.*, 2013 ▶); and visualization of lattice dynamics and material failure in shock-compressed matter (Milathianaki *et al.*, 2013 ▶).

(vi) Atomic physics: demonstration of the long-standing prediction of sum frequency generation using an optical laser and hard X-rays (Glover *et al.*, 2012 ▶).

(vii) Surface science: real-time observation of surface bond breaking using an X-ray laser (Dell’Angela *et al.*, 2013 ▶).

## 5. Facility access   

LCLS instruments are open to academia, industry, government agencies and research institutes worldwide for scientific investigations. There are two calls for proposals per year and external peer-review committees evaluate proposals based on scientific merit and instrument suitability. Access is without charge for users who intend to publish their results. Prospective users are encouraged to contact instrument staff members to learn more about the science and technical capabilities of the facility, as well as opportunities for collaboration.

## 6. Conclusion and perspectives   

After just five years of operation, it is remarkable to note the speed and intensity with which LCLS has become fully operational and delivered a very significant array of scientific results. This has required new approaches to facility operations, instrument design, detector development, measurement techniques and data analysis. It can clearly be seen that X-ray FELs should be considered as a new type of light source, avoiding inappropriate and misleading extrapolations from preceding light sources. The fourth-generation light sources are now clearly identified as diffraction-limited storage ring sources (Eriksson *et al.*, 2014 ▶) and will inevitably have a bright future. The scientific impact and the success of X-ray FEL facilities, going forward, will be determined by the degree to which the science opportunities can be derived from fundamentally new approaches to defining theoretical goals, experimental designs and data analysis.

Such opportunities have been dramatically strengthened by the emergence of other X-ray FEL facilities around the world: SACLA and SCSS in Japan (Ishikawa *et al.*, 2012 ▶; Shintake *et al.*, 2008 ▶), the European XFEL and FLASH in Germany (XFEL, 2006 ▶; Ayvazyan *et al.*, 2006 ▶), Swiss-FEL in Switzerland (Patterson *et al.*, 2014 ▶), PAL-XFEL in Korea (Nam *et al.*, 2013 ▶), FERMI in Italy (Allaria *et al.*, 2010 ▶), and most recently by the plans to deploy a major upgrade to the specifications of LCLS.

The LCLS-II project will provide a superconducting linac capable of producing a continuous stream of X-ray pulses at repetition rates of up to 1 MHz over a spectral range that extends from 250 eV to 5 keV. This will be built alongside the existing linac, whose continued operation at 120 Hz will be extended to provide high-energy pulses from 1 to 25 keV. This major upgrade will allow LCLS to continue leading scientifically for several decades to come, pushing the copper linac close to an unprecedented century of scientific service.
